# Male mice lacking ADAMTS-16 are fertile but exhibit testes of reduced weight

**DOI:** 10.1038/s41598-019-53900-0

**Published:** 2019-11-20

**Authors:** Catherine Livermore, Nick Warr, Nicolas Chalon, Pam Siggers, Joffrey Mianné, Gemma Codner, Lydia Teboul, Sara Wells, Andy Greenfield

**Affiliations:** 1 Mammalian Genetics Unit, Medical Research Council, Harwell Institute, Oxfordshire, OX11 0RD UK; 2 Mary Lyon Centre, Medical Research Council, Harwell Institute, Oxfordshire, OX11 0RD UK; 3 Present Address: Institute for Regenerative Medicine and Biotherapy, University of Montpellier, Institut National de la Santé et de la Recherche Médicale (INSERM), Centre Hospitalier Universitaire Montpellier, Montpellier, France

**Keywords:** Germline development, Organogenesis, Animal breeding

## Abstract

*Adamts16* encodes a disintegrin-like and metalloproteinase with thrombospondin motifs, 16, a member of a family of multi-domain, zinc-binding proteinases. ADAMTS-16 is implicated in a number of pathological conditions, including hypertension, cancer and osteoarthritis. A large number of observations, including a recent report of human *ADAMTS16* variants in cases of 46,XY disorders/differences of sex development (DSD), also implicate this gene in human testis determination. We used CRISPR/Cas9 genome editing to generate a loss-of-function allele in the mouse in order to examine whether ADAMTS-16 functions in mouse testis determination or testicular function. Male mice lacking *Adamts16* on the C57BL/6N background undergo normal testis determination in the fetal period. However, adult homozygotes have an average testis weight that is around 10% lower than age-matched controls. Cohorts of mutant males tested at 3-months and 6-months of age were fertile. We conclude that ADAMTS-16 is not required for testis determination or male fertility in mice. We discuss these phenotypic data and their significance for our understanding of ADAMTS-16 function.

## Introduction

ADAMTS (a disintegrin-like and metalloproteinase with thrombospondin motifs) proteins comprise a 19-member family of extracellular metalloproteinases implicated in a number of molecular processes, including collagen processing, proteoglycan cleavage and inhibition of angiogenesis^1^. Indeed, their control of the structure and function of the extracellular matrix (ECM) is thought to underpin diverse roles in tissue morphogenesis and patho-physiological remodelling^2^. ADAMTS-16 is a family member expressed in chondrocytes^3^. A number of observations also implicate ADAMTS-16 in reproductive function. It is expressed at high levels in human ovary^4^, where it is detected in the granulosa cells of pre-ovulatory follicles^5^. Its expression in mature follicles can be stimulated by follicle-stimulating hormone^5^.

In XY males, ADAMTS-16 is co-expressed with the gene encoding Wilms’ tumour protein 1, *Wt1*, in both embryonic kidneys and gonads, and in adult testes, suggesting a role in genitourinary development^6^. Indeed, a recent report describes the identification of three human *ADAMTS16* variants, resulting in amino acid substitutions predicted to be damaging, in cases of 46,XY disorders/differences of sex development (DSD)^7^. Two of these variants were identified in individuals with 46,XY complete gonadal dysgenesis i.e. XY female presentation. A third case had ambiguous genitalia at birth. No loss-of-function mouse model exists that allows the determination of ADAMTS-16’s physiological function, including any role in sex development. A rat model has been described; in addition to a role in blood pressure regulation^8^, disruption of *ADAMTS16* in rats causes male infertility and failure of testicular descent (cryptorchidism)^9^. The testes of homozygous mutant rats were significantly smaller than controls and histological examination revealed that the seminiferous tubules could not support spermatogenesis and progressively lost germ cells. In contrast, female rats lacking ADAMTS-16 were fertile.

In order to determine whether ADAMTS-16 plays a role in XY mouse reproductive biology, in particular sex determination and testicular function, we used CRISPR/Cas9 genome editing to introduce a 906 bp deletion into the *Adamts16* gene that removes exon 5. This deletion event removes part of the highly conserved protease domain of the enzyme and results in a predicted premature stop codon and nonsense-mediated decay. Homozygous XY mutants undergo normal primary sex determination and develop testes in the fetal period. However, adult mutant testes are smaller on average than those of controls. Nevertheless, all mutant males in two cohorts tested at 3 and 6 months of age had comparable fertility to controls. We discuss the significance of these data for our understanding of ADAMTS-16 function in reproduction.

## Results

### Analysis of *Adamts16* gene expression in the developing gonads

**Figure 1:**
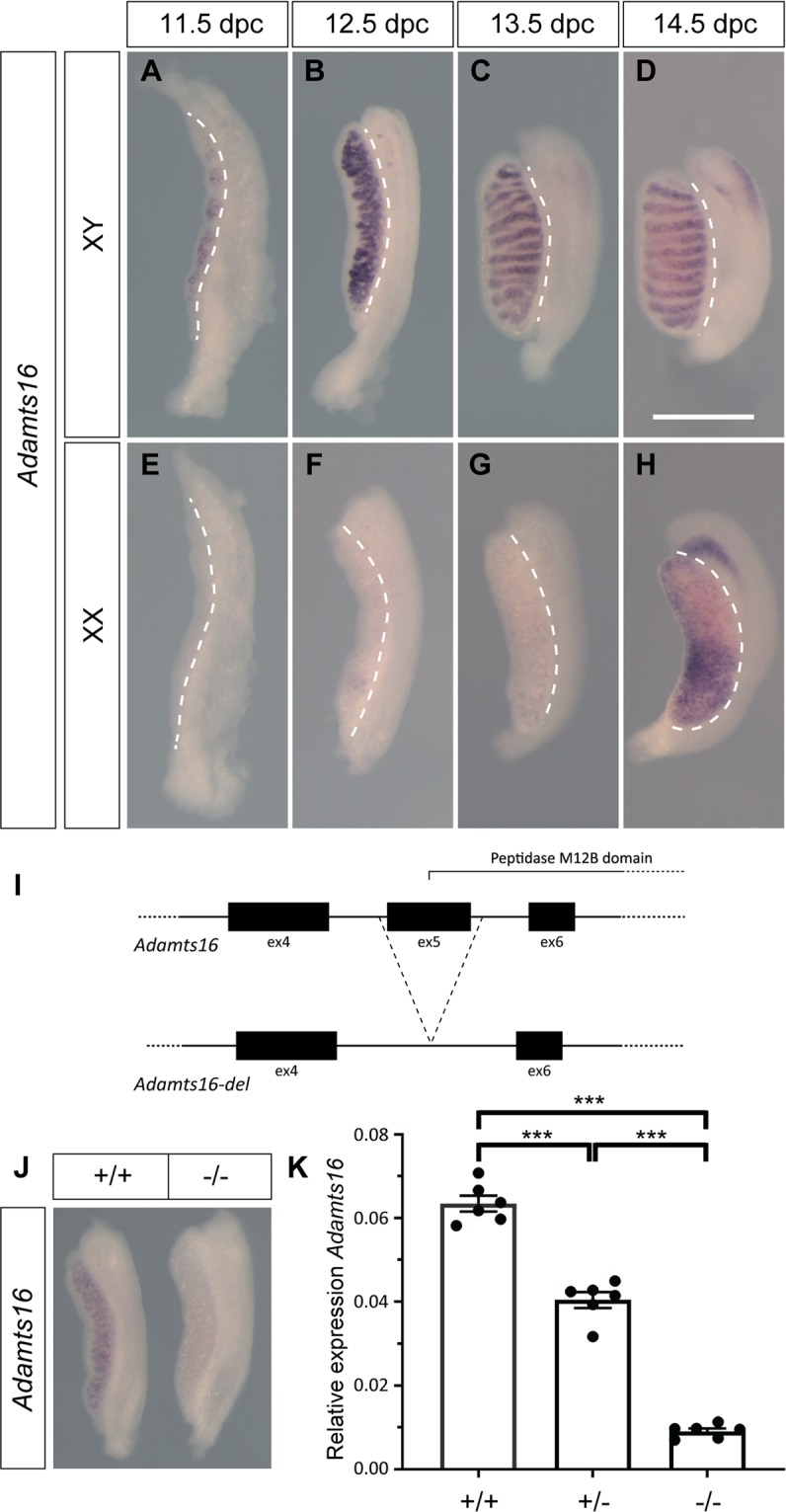
*Adamts16* expression and impact of targeted exon deletion. (**A**–**D**) Wholemount *in situ* hybridisation (WMISH) with *Adamts16* probe reveals expression at all developmental stages between 11.5 and 14.5 dpc in XY gonads. Expression is prominent in the testis cords (**B**–**D**); (**E**–**H**) Significant expression in XX gonads is detected only at 14.5 dpc (**H**); (**I**) Exon 5 of *Adamts16*, encoding part of the peptidase domain, was deleted using CRISPR/Cas9-mediated genome editing (not to scale). See Materials and Methods; (**J**) WMISH reveals *Adamts16* in control (+/+) gonad at 12.5 dpc, but negligible expression in the homozygous mutant (−/−) gonad at the same stage; signal in the mutant gonad was visible upon microscopic examination but was insufficient for photographic reproduction. (**K**) qRT-PCR shows dramatic reduction in *Adamts16* expression in homozygous mutant kidneys (−/−), and intermediate levels in heterozygotes (+/−). In all images in (**A**–**H**), the developing gonad is to the left of the white dotted line and the mesonephros is to the right. ***p ≤ 0.01 (student’s *t*-test).

We used wholemount *in situ* hybridisation (WMISH) and quantitative reverse transcriptase PCR (qRT-PCR) to examine *Adamts16* expression in the developing gonads and reproductive tracts, between 11.5 and 14.5 dpc (Fig. 1). Expression was detected by WMISH in XY gonads at all stages from 11.5 dpc, and between 12.5 and 14.5 dpc this was prominent in the cords of the developing testis in a profile consistent with Sertoli cell expression (Fig. 1A–D). In contrast, significant signal in the XX gonads was not detected until 14.5 dpc (Fig. 1E–H). Sexually dimorphic expression was confirmed by qRT-PCR of gonadal tissue at 12.5 dpc (Fig. S1). These data, including the observed expression in the supporting cell lineage at the time of sex determination, are consistent with those generated previously by microarray-based expression profiling and single-cell RNA sequencing^10,11^.

### Generation and characterisation of an *Adamts16* loss-of-function allele using CRISPR/Cas9

We sought to use genome editing to generate a mouse *Adamts16* loss-of-function model on a defined genetic background to permit careful analysis of the role of ADAMTS-16 in sex determination and testis development. We chose C57BL/6N as a strain background because, like the closely related C57BL/6J, it is sensitised to disruptions to testis determination^12–15^. The *Adamts16* gene comprises 23 exons. In order to disrupt *Adamts16* we used microinjection of Cas9 RNA/guide RNAs into 1-cell C57BL/6Ntac embryos to target and excise exon 5, which encodes part of the highly conserved, zinc-dependent metalloprotease domain (see Fig. 1I and Methods). We identified founders harbouring two deletions of this exon, one of 906 nucleotides (line 1) and one of 923 nucleotides (line 2). Both alleles result in a complete loss of exon 5; splicing from exon 4 to exon 6 results in a predicted frame-shift and early chain termination due to a premature stop codon. Both alleles were transmitted to the next generation following breeding. We focus here on line 1, but equivalent data were obtained for line 2.

### Analysis of gonad development in XY embryos lacking functional ADAMTS16 reveals no overt abnormalities

Male and female *Adamts16* heterozygotes were normal and fertile. We generated homozygous mutants by inter-crossing heterozygotes. Semi-quantitative analysis of *Adamts16* expression in the homozygous XY mutant gonad at 12.5 dpc showed a dramatic reduction in signal when compared to controls (Fig. 1J). To confirm this loss of transcript in the mutant, we carefully quantified *Adamts16* expression in control and homozygous mutant kidneys, a known site of expression, using qRT-PCR (Fig. 1K). This analysis revealed robust wild-type expression, negligible expression in homozygous mutants, and intermediate levels in heterozygotes, possibly due to nonsense-mediated decay of the mutated mRNA transcript. Given this significantly reduced expression of the mutant allele, and the predicted disruption to functionality of the ADAMTS-16 protein encoded by it, which lacks a portion of the critical protease domain, we conclude that the edited *Adamts16* variant is a null allele or a severely hypomorphic allele.

**Figure 2:**
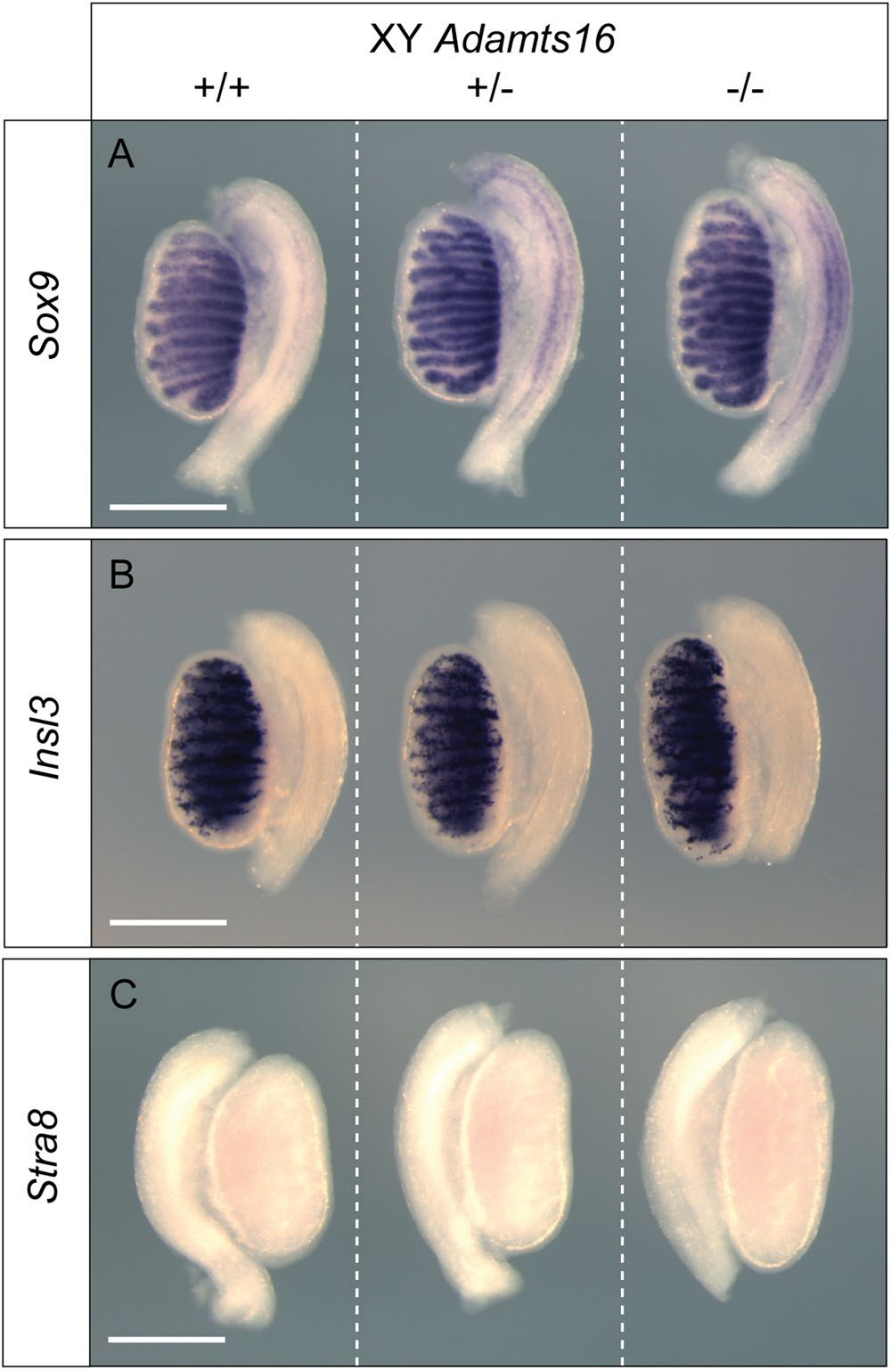
Marker gene analysis of XY gonad development in *Adamts16* homozygous mutants. Expression of the Sertoli cell marker *Sox9* (**A**), Leydig cell marker *Insl3* (**B**) and the germ cell meiotic entry marker *Stra8* are all unaffected in *Adamts16* homozygous mutants (−/−) when compared to wild-type (+/+) and heterozygous (+/−) controls at 14.5 dpc. Scale bar = 0.5 mm.

Given the testicular phenotype of rats lacking ADAMTS-16, we examined testis morphology and marker gene expression in homozygous mutants and controls at 14.5 dpc, a fetal stage at which sex determination is essentially complete. Wholemount *in situ* hybridisation (WMISH) analysis with *Sox9*, a key testis-determining gene and marker of Sertoli cells^16^, revealed testis cord formation in both mutant and control gonads, which were of comparable size (Fig. 2). There was no evidence of any disruption to testis cord morphology. Similarly, WMISH with *Insl3*, a marker of fetal Leydig cells, also revealed no overt abnormalities in mutant gonads (Fig. 2). Finally, WMISH with *Stra8*, which marks germ cells entering meiosis that are normally only found in ovaries at this stage, revealed no evidence of any feminisation of mutant XY gonads. From these data showing that somatic and germ cell development proceeds normally during testis development in XY mutant gonads, we conclude that ADAMTS-16 is not required for testis determination in mice. An examination of marker gene expression in XX mutant gonads at the same developmental stage also revealed no overt abnormalities (Fig. S2).

### Normal fertility in adult males lacking ADAMTS-16

Homozygous mutant animals were viable and appeared normal at weaning and later stages of adult life. Cohorts of homozygous mutant and control males were tested for fertility at 3 months and 6 months of age (see Materials and Methods). No significant differences were observed between mutant and control cohorts in respect of their ability to generate copulatory plugs in wild-type females and give rise to fetuses at 13.5 to 16.5 dpc (Fig. S3). Homozygous female mice were similarly fertile and exhibited no overt morphological abnormalities of the reproductive system (data not shown).

**Figure 3:**
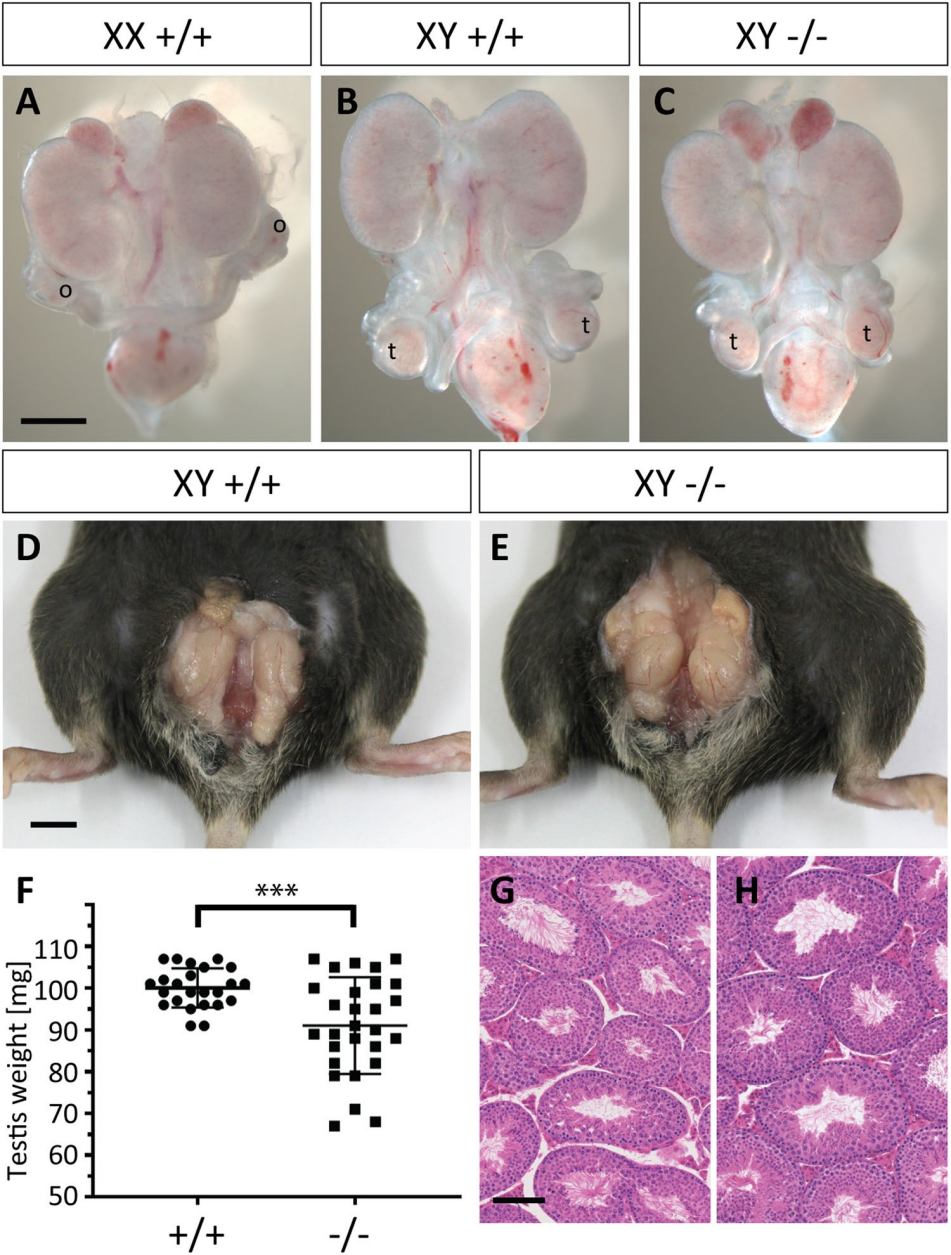
Fetal and adult reproductive structures are overtly normal in *Adamts16*-deficient mice. (**A**–**C**) At 16.5 dpc, the fetal testes (t) have descended to a position adjacent to the bladder in wild-type (**B**) and *Adamts16* −/− mutant males (**C**); this is in contrast to the para-renal position of the ovaries (o) in wild-type females at the same stage (**A**). Scale bar = 0.5 mm; (**D**,**E**) Testes in adult mice have descended in to the scrotum in wild-type (**D**) and *Adamts16* −/− mutant males (**E**); scale bar = 0.5 cm; (**F**) The average weight of the adult testis is reduced in the *Adamts16* −/− mutant males (n = 28) compared to wild-type controls (n = 24); ***p ≤ 0.01 (student’s *t*-test); (**G**,**H**) Testicular histology of H&E-stained section appears unaltered in mutant males (**H**) when compared to wild-type controls (**G**). Scale bar = 100 μm.

In rats lacking ADAMTS-16, testes appeared high in the abdomen, potentially indicating a defect in the first, trans-abdominal, phase of testicular descent^9^. We examined testicular position in mutants and controls at 16.5 dpc, by which time the transabdominal phase is essentially complete. Testes in both cohorts were positioned at the base of the abdomen (Fig. 3A–C), indicating that the trans-abdominal phase of testicular descent has occurred normally in ADAMTS-16 deficient mice. Consistent with this observation, *Adamts16* expression was negligible in the gubernaculum at 14.5 dpc, when compared to its expression in testis and expression of the gubernacular marker gene, *Lgr8* (Fig. S4). Following fertility testing, adult mutant males and controls were sacrificed and the testes examined. There were no overt differences between the positions of testes in mutants or controls (Fig. 3D,E); in both cases, the testes were commonly scrotal but occasionally found in a supra-scrotal position, as is common in mice. There was a small but significant reduction (9%) in average testis weight in mutants (Fig. 3F). We also examined the histology of control and mutant testes. Consistent with their normal fertility, the testes of mutant males also appeared overtly normal (Fig. 3G,H).

## Discussion

Here, we describe the generation of an *Adamts16* mutant allele through CRISPR/Cas9-mediated genome editing. *Adamts16* transcript levels are negligible in homozygous mutant embryonic tissue, probably as a consequence of nonsense-mediated decay of the mutant transcript. Thus, the mutant allele described here is likely a null allele or a strongly hypomorphic allele. Homozygous XY male mice lacking functional ADAMTS-16 undergo normal testis determination. The testes of adult mutant males are somewhat smaller than controls but they exhibit normal histology. These observations are consistent with the fertility of homozygous male mutants, at 3 and 6 months of age. We conclude that ADAMTS-16 is not required for testis development in mice.

Several prior observations indicated a possible role for ADAMTS-16 in gonadal sex determination. First, *Adamts16* transcripts are detected in the developing mouse gonad at the stage of sex determination and have been reported to be at higher levels in XY supporting cells (pre-Sertoli cells) than XX pre-granulosa cells^10^. Our WMISH data confirm male-enhanced expression of *Adamts16* in the gonad at 11.5 dpc, the sex-determining stage of gonadogenesis; such sexual dimorphism in gene expression sometimes reflects a role in testis determination. Secondly, the *Adamts16* locus is bound by SRY and SOX9 based on genome-wide ChIP-seq analyses of those transcription factors in the developing testis^17^, suggesting that it may be a regulatory target of these core testis-determining factors. *Adamts16* has also been reported as a potential target of WT1^6^, a transcription factor with known roles in sex determination. Finally, mis-sense variants of *ADAMTS16*, predicted to be damaging, are associated with human 46,XY DSD, including complete gonadal dysgenesis (female presentation), indicating a possible role in human testis determination^7^. However, such a role is not supported by the overtly normal sex determination in XY and XX mouse fetuses reported here. In this context, it is interesting to note that a large number of *Adamts* genes are detected in the developing gonad at the stage of sex determination^10^, suggesting the possibility of functional redundancy. Whilst it is not uncommon for a mouse knockout not to phenotypically model a human heterozygous variant associated with disease, we cannot exclude the possibility that *ADAMTS16* variants associated with DSD are mere polymorphisms or, alternatively, not loss-of-function alleles, but rather gain-of-function or another class of disruptive allele.

Male rats lacking functional ADAMTS-16 exhibited significantly smaller testes than controls, with more pronounced differences as the rats aged^9^. Moreover, mutant males were infertile and bilaterally cryptorchid. One outstanding question concerns why the phenotype associated with loss of ADAMTS-16 in rats is much more severe than that detected in mice. Such questions can always be answered with reference to species-specific differences in particular biological processes: different genomes, different gene expression levels and temporal profiles, and different tissues and contexts. One other explanation might concern the alleles generated in rat and mice: are they both loss-of-function alleles? Expression analyses reported here indicate that in mouse mutants homozygous for the induced *Adamts16* mutation there is significant loss of *Adamts16* mRNA, this in addition to the loss of exon 5-encoded amino acids and predicted introduction of a premature stop codon. The rat mutation comprised a 17 base-pair deletion in exon 1, introducing a predicted premature stop codon and thus also constituting a likely null allele^9^. The modest alteration in testis weight observed in the homozygous mutant male mice is consistent with the absence of a cryptorchidism phenotype. The difference in phenotype between rat and mouse remains unexplained, but the species-specific compensatory action of other ADAMTS proteins may be a contributory factor. We did not observe significant expression of *Adamts16* in the fetal mouse gubernaculum, a structure that controls testicular descent by undergoing significant remodelling during this period; it is unclear whether it is expressed in the developing rat gubernaculum.

The variability in testis weight of mutant males represents an example of phenotypic variation on a pure-bred genetic background. Whilst the mechanistic basis of such reduced penetrance, or variable expressivity, remains unclear it is not an uncommon observation even when little or no genetic variation exists between animals in a cohort. A recent report of a high-throughput study of mouse knockout phenotypes, performed on a cohort of isogenic mice, indicates that variable expressivity is widespread^18^. Stochastic influences on the severity of sex development phenotypes are also common^19^.

In summary, we report normal sexual development in male mice lacking functional ADAMTS-16. Since C57BL/6 comprises genetic backgrounds sensitised to disruptions to testis determination, it seems most unlikely that any sex development phenotypes would become apparent on any other mouse inbred strains. Therefore, we conclude that ADAMTS-16 is dispensable for testis determination and testicular function in mice, despite a wealth of data suggesting a potential role for this protein in sexual development.

## Methods

### Mouse strains

The Animal Welfare and Ethical Review Body (AWERB) at MRC Harwell approved the mouse experiments reported here. All mice were bred under license from the UK Home Office (PPL 70/8898) and experiments performed according to the Animals (Scientific Procedures) Act, 1986. Mice were kept in individually ventilated cages (IVCs) in an environment that was free of specific opportunistic pathogens (SPF). Further details of micro- and macro-environmental conditions are available upon request. Mice harbouring a 906 bp deletion of *Adamts16* were generated using CRISPR/Cas9. Two pairs of single-guide RNAs (gRNAs) flanking ENSMUSE00000448614, along with Cas9 RNA, were injected into 1-cell mouse zygotes from C57BL6/NTac (B6/N) females, using methodologies as previously described^20,21^. gRNA pair sequences, including protospacer adjacent motifs (PAM), were: 5.1/5.2-TATATGATGCTCGTACAATC(TGG), CTCGTACAATCTGGATGTCT(TGG) and 3.1/3.2-AAGACTCAGCTACACAAATG(AGG), TCACACACTAGAAATCTCAT(TGG).

All lines were maintained on B6/N. Genotyping of *Adamts16* mice was performed using a common forward primer (5′-GAGTCCTGCTCTGCAAGTCC-3′) and two reverse primers that distinguish between the wild-type and deletion alleles: 5′-AGGCATGTCTATGGAGGAACAT-3′ (wild-type) and 5′-TGTGTGAAGATATCAGTGAGCCC-3′ (deletion).

### Generation of embryos

Noon on the day the copulatory plug was detected was counted as 0.5 days *post coitum* (dpc). Adult mice were sacrificed in humane fashion by dislocating the neck, and this was confirmed by cessation of circulation; embryos were decapitated in ice-cold, phosphate buffered saline (PBS). Accurate staging of embryos collected at 11.5 dpc was performed by carefully counting the number of tail somites (ts).

### Wholemount *in situ* hybridization

Wholemount *in situ* hybridization (WMISH) of embryonic tissues, and the use of probes for the genes *Sox9*, *Stra8*, *Insl3* and *Lgr8*, have been described previously^22,23^. A 566 bp *Adamts16* probe was generated by PCR amplification of fetal kidney cDNA using primers 5′-GGCAACATTATGTCCCCAAC-3′ and 5′-TCCAGAGGGCATCTCTGACT-3′. At least three independent biological samples (per genotype) were analysed with a given marker.

### Quantitative RT-PCR

Total gonadal RNA (following removal of the mesonephros) was extracted using the RNeasy plus micro kit (Qiagen). Reverse transcription (RT) was performed using 100 ng of total RNA, using the High capacity cDNA RT kit (Applied 24 Biosystem). Quantitative RT-PCR (qRT-PCR) was with Fast SYBR Green Master Mix (Life technologies), using a 7500 Fast Real-Time PCR system (Applied Biosystem). RNA expression levels were calculated following normalization to those of *Hprt1* (endogenous control), employing the ΔΔCt method. At least five samples per genotype were included in the analysis. Sequences of primers are available on request.

## Supplementary information

Supplementary Figures
