# Supplementary material for: Male mice lacking ADAMTS-16 are fertile but exhibit testes of reduced weight
Source: Sci Rep. 2019 Nov 20;9:17195. doi: 10.1038/s41598-019-53900-0 (PMC6868159; doi:10.1038/s41598-019-53900-0)
Supplement: Supplementary file 1 — Supplementary Figures [file 41598_2019_53900_MOESM1_ESM.pdf]

SUPPLEMENTARY FIGURES

for

Male mice lacking ADAMTS-16 are fertile but exhibit testes of reduced weight

by

Catherine Livermore, Nick Warr, Nicolas Chalon, Pam Siggers, Joffrey Mianné, Gemma Codner, Lydia Teboul, Sara Wells & Andy Greenfield

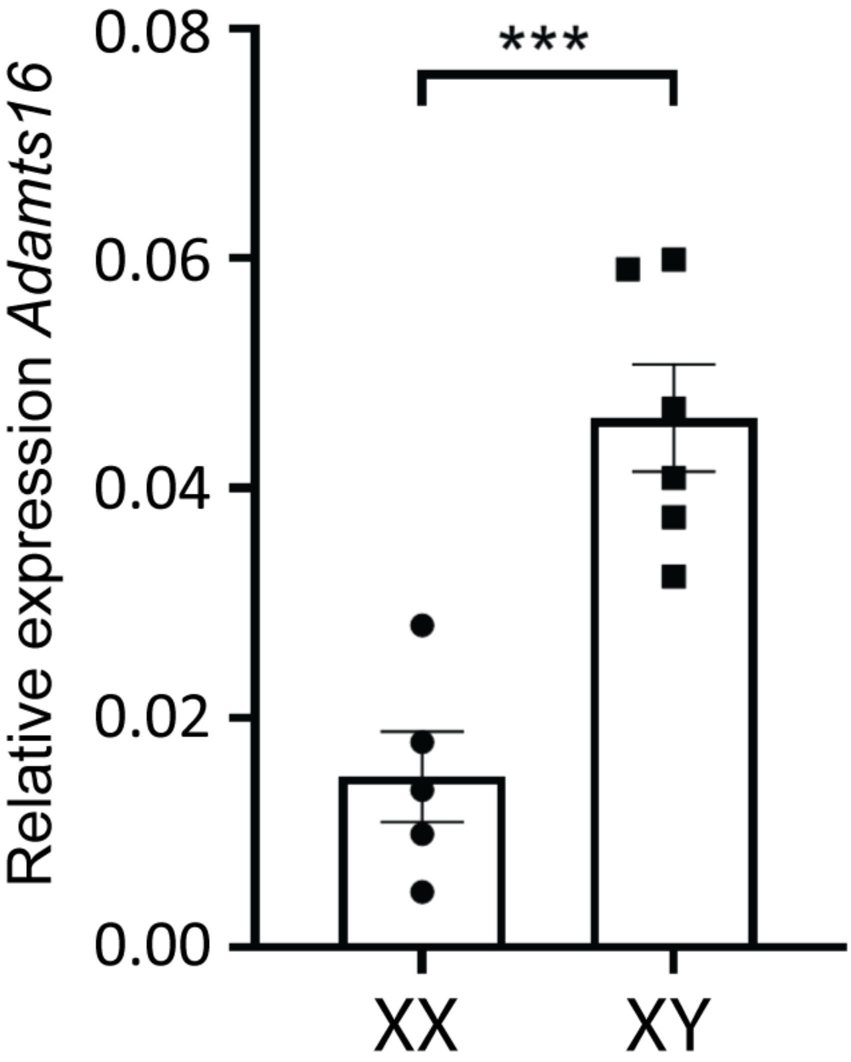

Figure S1. Analysis of expression of *Adamts16* expression in the developing gonads of female (XX) and male (XY) embryos at 12.5 dpc using qRT-PCR. ***, $p \leq 0.01$ (student's *t*-test).

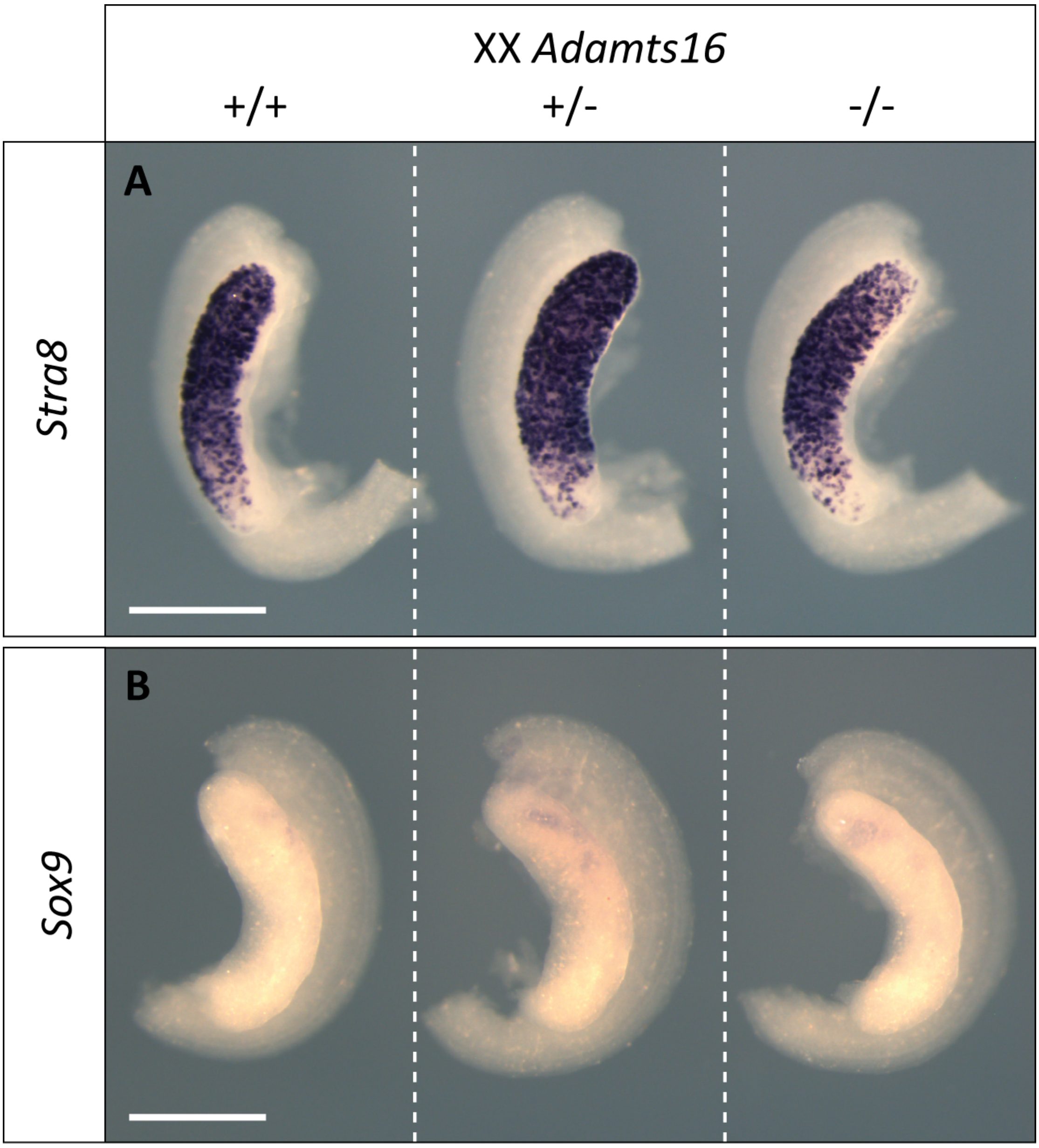

Figure S2. Marker gene expression in XX control and mutant gonads at 14.5 dpc. A) Expression of the germ cell marker *Stra8* is unaffected in *Adamts16* -/- XX gonads at 14.5 dpc when compared to heterozygous and wild-type controls; B) Similarly, *Sox9* expression is unaffected in homozygous mutants compared to controls. Scale bar = 0.5 mm.

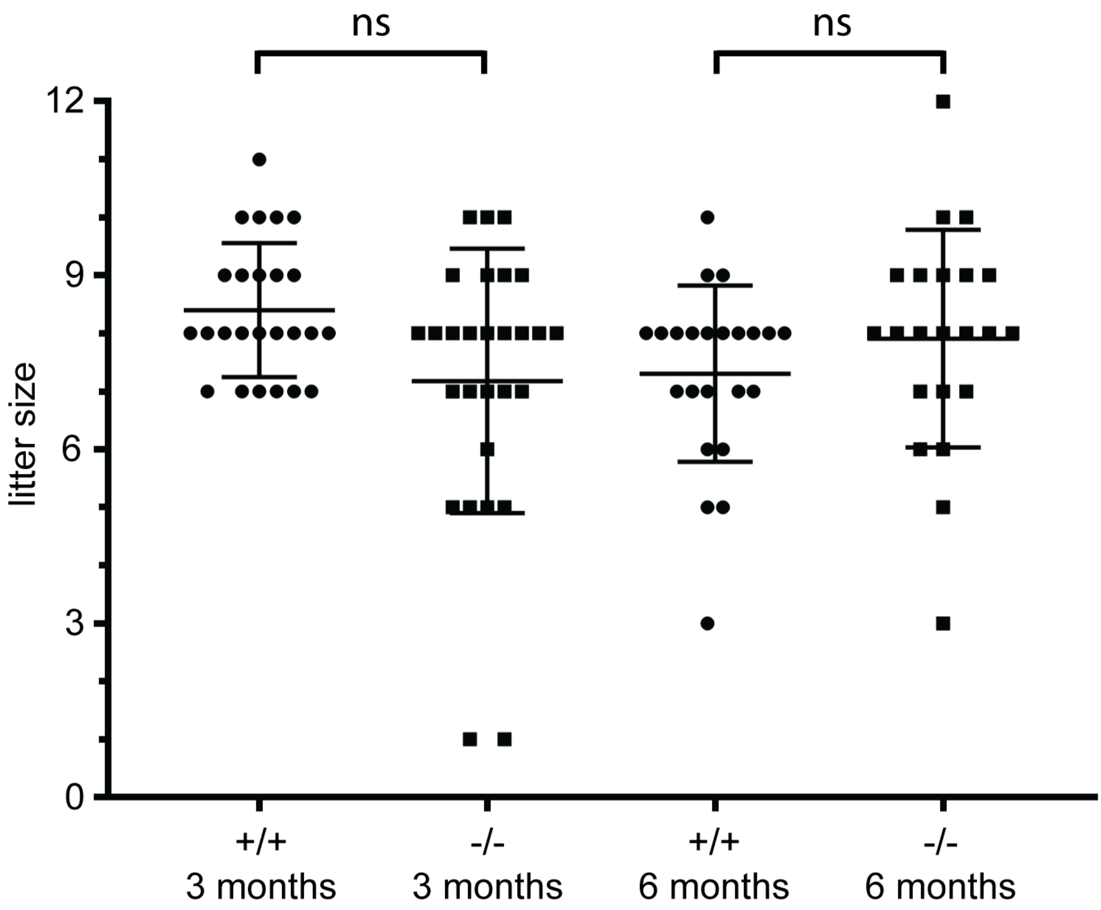

Figure S3. Normal fertility of males lacking ADAMTS-16. Wild-type control (+/+) and *Adamts16* homozygous mutant (-/-) males were tested at two distinct ages for their ability to generate fetuses following matings with wild-type control females. At 3 months of age, the difference in average size of litters from control (n=25) and mutant (n=28) males was not significant (ns; $p = 0.556$, two-tailed Mann-Whitney test). Nor was a significant difference detected in average litter sizes at 6 months of age (n= 23 controls, n= 22 mutants; $p = 0.1772$).

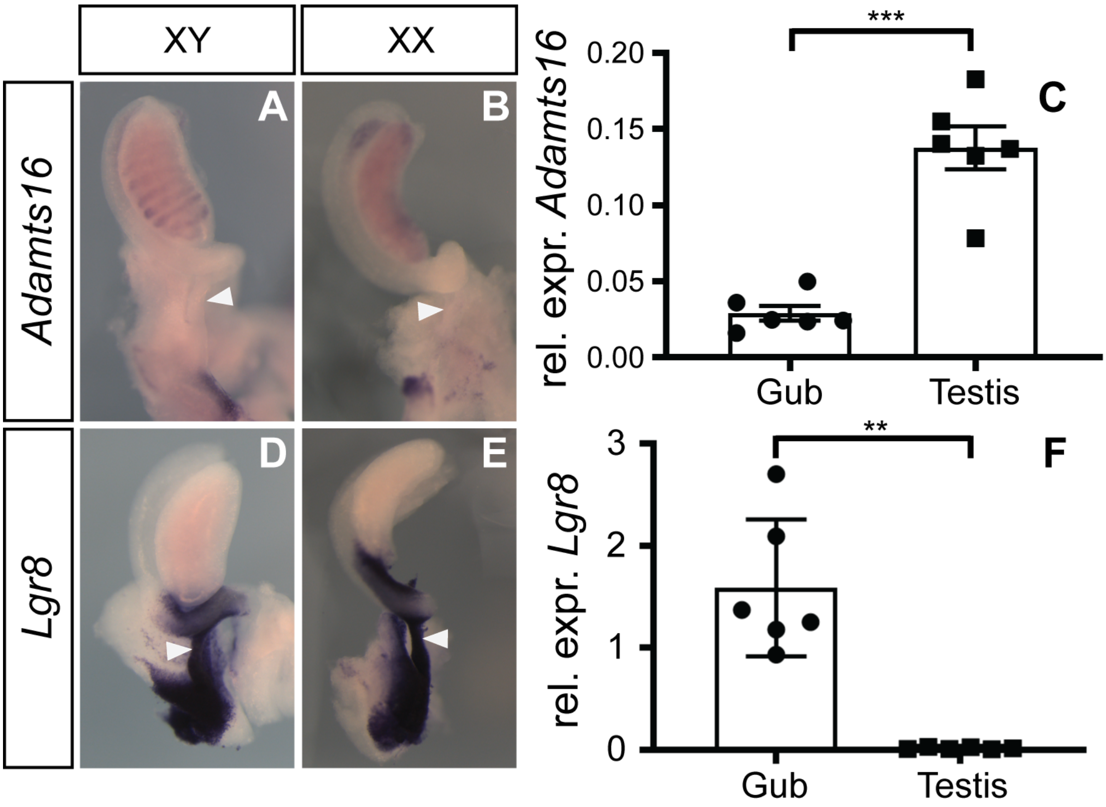

Figure S4. Analysis of *Adamts16* expression in the fetal gubernaculum at 14.5 dpc. A-C) WMISH with an *Adamts16* probe reveals expression in the testis cords of the XY gonad (A), but no detectable expression in the gubernaculum (white arrowhead) at the same stage. Expression is also undetectable in the XX gubernaculum (B); qRT-PCR reveals negligible expression in the gubernaculum (Gub) compared to that in the testis (C); D-F) Control experiments, examining expression of the known gubernaculum marker *Lgr8,* reveal strong expression in the gubernaculum of XY (D) and XX (E) fetuses (white arrowheads), confirmed by qRT-PCR (F). *Lgr8* is undetectable in the gonads at this stage (D-F). ***, $p \leq 0.01$; **, $p \leq 0.05$ (student's *t*-test).
